# Should Any Workplace Be Exempt from Smoke-Free Law: The Irish Experience

**DOI:** 10.1155/2012/545483

**Published:** 2012-05-24

**Authors:** M. McCaffrey, P. Goodman, A. Gavigan, C. Kenny, C. Hogg, L. Byrne, J. McLaughlin, K. Young, L. Clancy

**Affiliations:** ^1^School of Physics, Dublin Institute of Technology, Dublin 8, Ireland; ^2^Environmental Health Department, Health Service Executive, Dublin 15, Ireland; ^3^Tobacco Free Research Institute, Digital Depot, Dublin 8, Ireland; ^4^School of Physics, University College Dublin, Dublin 4, Ireland

## Abstract

*Background*. In 2004, the Irish Government introduced national legislation banning smoking in workplaces; with exemptions for “a place of residence”. This paper summarises three Irish studies of exempted premises; prisons, psychiatric hospitals and nursing homes. *Methods*. PM_2.5_ and nicotine were measured in nursing homes and psychiatric hospitals, in addition to ultrafine particles in the hospitals. In the prisons, officers (*n* = 30) completed exhaled breath Carbon Monoxide (CO) measurements. Questionnaires determined officers' opinion on introducing smoking prohibitions in prisons. Nursing home smoking policies were examined and questionnaires completed by staff regarding workplace secondhand smoke (SHS) exposure. *Findings*. Ultrafine particle concentrations in psychiatric hospitals averaged 130,000  cm^3^, approximately 45% higher than Dublin pub (35.5 **μ**g/m^3^) pre ban. PM_2.5 _ levels in psychiatric hospitals (39.5 *μ*g/m^3^) were similar to Dublin pubs (35.5 *μ*g/m^3^) pre ban. In nursing homes permitting smoking, similar PM_2.5 _ levels (33 **μ**g/m^3^) were measured, with nicotine levels (0.57 **μ**g/m^3^) four times higher than “non-smoking” nursing homes (0.13 **μ**g/m^3^). In prisons, 44% of non-smoking officers exhibited exhaled breath CO criteria for light to heavy smokers. *Conclusions*. With SHS exposure levels in some exempted workplaces similar to Dublin pubs levels pre ban, policies ensuring full protection must be developed and implemented as a right for workers, inmates and patients.

## 1. Introduction

Environmental tobacco smoke (ETS) has been associated with increases in rates of cancer, respiratory, and cardiovascular disease. In pursuance of a policy to create a tobacco free society the Irish Government on the 29th March 2004 introduced the first national comprehensive legislation banning smoking in all workplaces. It was introduced as a public health measure to offer protection to workers, and the public who are exposed to the harmful and toxic effects of ETS in workplaces. However, a number of exemptions were permitted, such as prisons, psychiatric hospitals, and long-term residential care institutions including nursing homes, on the premise that such locations were deemed the occupiers' home.

Whilst some areas have been exempted from the Irish Smoke-free Workplace Legislation, it is important to bear in mind that all employers still have the right to enforce the Legislation and every employer has a duty of care to an employee under the Safety, Health and Welfare at Work Act 2005. The exemption only confers the right not to be penalised for nonenforcement.

This paper presents an overview of three individual Irish studies conducted in selected exempted premises, namely, prisons, psychiatric hospitals, and nursing homes.

Prisons, psychiatric hospitals, and nursing homes are not only a place of work, but also a place of residence for inmates and residents. There is currently no law in Ireland to prevent people from smoking in their own homes, so is it justified to take this away from smokers in their place of residence? Conversely, is it right that staff are surrounded by polluted air which can affect their health, with nonsmoker inmates and residents unable to avoid the harmful effects of secondhand smoke around them.

## 2. Equipment Used

The concentrations of ultrafine particles were determined using a handheld automatic particle counter (TSI P-Trak). Ultrafine particles, in the size range of 10^−9^ to 10^−6 ^m, are good indicators of tobacco smoke and have been postulated as the size which is capable of quick uptake in the blood, thus likely to influence acute and chronic health effects [1, 2]. The mass of the larger airborne particles (PM_2.5_ and PM_10_) was collected using a handheld automatic particle counter (METONE Aerocet-531). Ambient carbon monoxide (CO) gas levels were recorded using a hand-held monitor (TSI Q-Trak). Carbon monoxide (CO) is a colorless, odorless gas that is produced as a result of incomplete burning of carbon-containing materials, such as tobacco, thus a very important metric to quantify. Breath carbon monoxide levels were measured using a Smoke Check carbon monoxide monitor. Nicotine was assessed using passive nicotine absorbers with filter paper treated with sodium bisulphate to which nicotine binds. The filter paper was analysed using an approved protocol in one of Europe's leading laboratories (Servei d'Avaluació i Mètodes d'Intervenció, Agència de Salut Pública de Barcelona, Spain). Nicotine is unique to tobacco smoke and therefore a good indicator of the presence of SHS.

## 3. Prisons Methodology

Logistically it was not possible for us to monitor PM_2.5_ or ultrafine particle levels in the prisons due to issues of equipment safety, and so information on the exposure of prison staff was obtained by a questionnaire completed by prison officers regarding their opinion on smoking in prisons. Unfortunately a questionnaire only provides subjective data, so additionally a number of prison officers (*n* = 30) completed a study of their exhaled breath CO levels.

Of the prison officers who completed the questionnaire (*n* = 90), 81% were males (of which 30% were smokers) and 19% were females (of which 47% were smokers).

## 4. Prison Results

**Figure 1:**
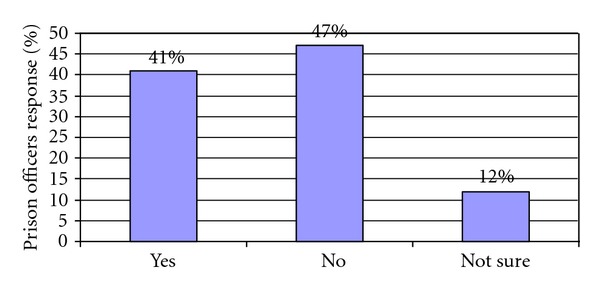
Should there be a smoking ban in prisons?

When asked should there be a complete smoking ban in prisons, 47% of prison officer respondents disagreed with a smoking ban being introduced (the majority of these were smokers themselves). Only 41% agreed with a smoking ban being introduced, the majority of them being nonsmokers (Figure 1).

**Figure 2:**
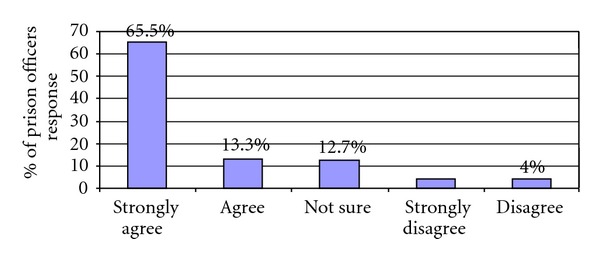
Should smoking be prohibited in all enclosed areas for example, cells, landings, halls, and recreational areas?

When asked if smoking should be prohibited in all enclosed areas within the prison, including designated no-smoking areas, for example, cells, halls, landings, and recreational areas (Figure 2), 79% of prison officers surveyed either strongly agreed (65.5%) or agreed (13.3%) that this should be the case.

**Figure 3:**
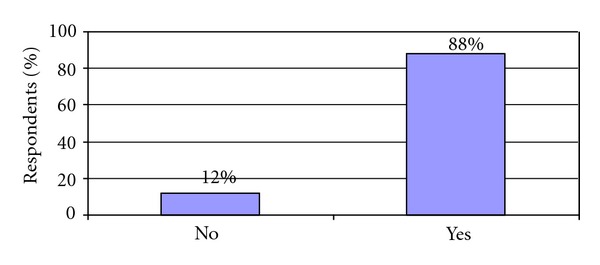
Would a complete smoking ban create more problems in the prison?

When asked if an outright smoking ban would create more problems within the prison, potentially causing an increase in behavioural problems, gang violence, and drug trafficking, 88% of respondents (Figure 3) believed that such problems would arise with an outright smoking ban. A small number of respondents gave other examples such as an increase in disturbances leading to riot and mental health issues such as anxiety.

### 4.1. Breath Carbon Monoxide Levels

**Figure 4:**
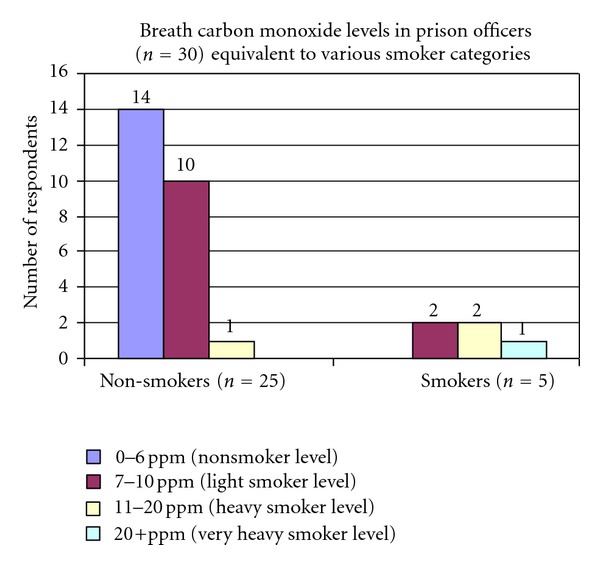
Number of nonsmoking prison officers (*n* = 25) with exhaled CO categorised into expected levels in smokers.

Breath carbon monoxide levels were measured in 25 nonsmoker and 5 smoker prison officers using a Smoke Check Carbon Monoxide monitor (Figure 4). Fourteen of the 25 nonsmokers had levels between 0 and 6 ppm which is categorised as a nonsmoker. Ten nonsmoking officers had CO levels between 7 and 10 ppm, which is categorised as a light smoker, while one nonsmoker had CO levels between 11 and 20 ppm which categorised him as a heavy smoker. Overall 44% of the 25 nonsmoking prison officers were categorised as being light-to-heavy smokers, of these one-third had home exposure, indicating that the majority were solely exposed to SHS in the workplace.

Of the 5 prison officers who were smokers, 2 had levels between 7 and 10 ppm, 2 had levels between 11 and 20 ppm, and 1 had a level of 20+ ppm.

## 5. Psychiatric Hospitals Methodology

In 6 psychiatric hospitals, located in both inner city and large country town settings, exposure measurements of ultrafine particles (TSI P-Trak) and PM_2.5_ (Metone Aerocet 531) were made for a period of 2-3 hours, to quantify the airborne particles in designated “smoking areas.” Ambient carbon monoxide (CO) gas levels were recorded in each location. Nicotine levels present inside and outside “smoking rooms” were also assessed. In conjunction with these measurements, the number of residents present within the “smoking area” and the number smoking were also recorded.

## 6. Psychiatric Hospitals Results

In the 6 psychiatric hospitals (Table 1), the ultrafine airborne particle concentrations peaked at approximately 400,000 per cm^3^ with an overall average of 130,000 per cm^3^. Within this small sample size, there was a large range of particle concentrations between hospitals, as much as a 6-fold difference in some instances. However, 2 of the hospitals (nos. 2 and 6) had specific outdoor structures for the smoking patients, which significantly lowered the exposure levels within the hospitals.

Particulate mass PM_2.5_ levels averaged 39.5 *μ*g/m^3^. Carbon monoxide levels averaged 3.87 ppm, while the available nicotine results averaged extremely high levels of 93.17 *μ*g/m^3^.

## 7. Nursing Homes Methodology

Twenty nursing homes in the County Meath/Kildare area were studied to determine particulate mass (PM_2.5_) and nicotine exposure levels using the same techniques as previously described. Questionnaires were also administered to staff regarding SHS exposure in their workplace and the smoking policies which were in place in their respective nursing homes.

## 8. Nursing Homes Results

**Figure 5:**
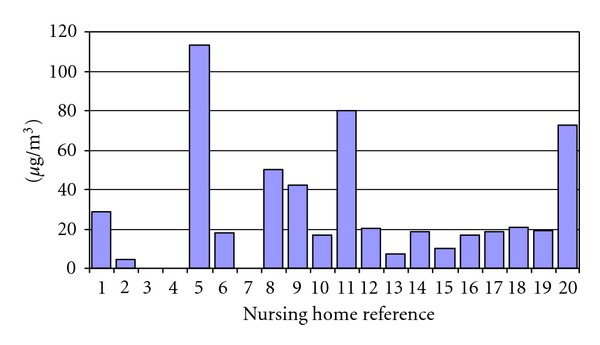
Average PM_2.5_ level in each nursing home smoking area. Nursing homes nos. 1 and 2 were nonsmoking control nursing homes. PM_2.5_ results were not available for nursing home smoking area nos. 3 and 4.

PM_2.5_ levels in nursing homes with smoking areas were approximately 8 times higher than nursing homes nos. 1 and 2, where smoking was completely prohibited (Figure 5). The PM_2.5_ levels in “smoking” nursing homes averaged 33 *μ*g/m^3^.

**Figure 6:**
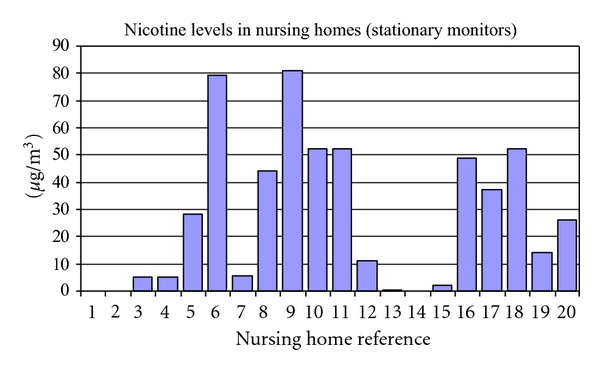
Nicotine levels indicated by stationary badges placed in the smoking areas of nursing homes. Nursing homes nos. 1 and 2 were nonsmoking control nursing homes.

In Figure 6, the average nicotine level in nursing home smoking areas was 27.3 *μ*g/m^3^.

**Figure 7:**
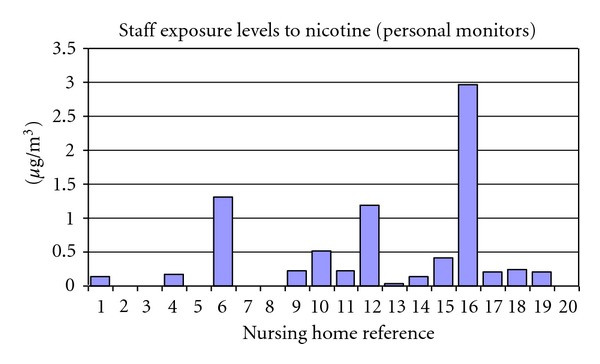
Staff exposure levels to nicotine indicated by personal badges worn during their work shift. Nursing homes nos. 1 and 2 were nonsmoking control nursing homes. Personal monitor results were not available for nursing home nos. 2, 3, 5, 7, and 20 due to issues with the filter paper. Nursing home no. 8 had very low staff exposure levels of <0.02 *μ*g/m^3^.

Figure 7 shows the personal nicotine exposure of staff during a work shift, measured using a portable badge attached to their uniform. Staff working in “smoking” nursing homes were exposed to average nicotine levels (0.57 *μ*g/m^3^) that were four times higher than the exposure of staff in the “nonsmoking” nursing homes (0.13 *μ*g/m^3^), (homes Nos. 1 and 2).

From the questionnaire survey of nursing home staff, 48% stated that SHS exposure in their workplace caused irritation.

A smoking rate of 7.5% was noted among residents of nursing homes surveyed, well below the national average. Individual nursing homes had differing policies in relation to smoking: two banned smoking completely, with the residents being made aware of this at registration stage. Nicotine replacement therapy was offered to aid their fight to quit tobacco should they agree to move into the home. Two homes permitted smoking anywhere indoors. The remainder of the nursing homes restricted smoking to a dedicated room, clearly marked as the smoking room and only the residents were permitted to smoke within. This room was segregated from the rest of the premises by a door leading into the main corridors of the premises, with some of these doors being self-closing. This method of segregating smokers from the rest of the building is in keeping with the policy on smoking in Care Homes in Northern Ireland [3]. 

## 9. Discussion

The results from these studies should inform policies to help minimise or eliminate the exposure of nonsmokers to tobacco smoke in exempt premises.

Prisons are seen as sensitive workplaces where every attempt to pacify inmates and protect workers is taken and as such require special attention when implementing a no-smoking policy. Tobacco use can be seen as an integral part of prison life and prison culture. It serves a range of functions including a means of social control, as a surrogate currency, as a symbol of freedom and in a group with few rights and privileges, a stress reliever and a social lubricant [4]. Smoking prevalence is much higher among the prisoners than the general population. The General Health Care Study of the Irish Prison Population in 2000, estimated that 91% of men were current smokers and up to 100% of women were smokers. A letter published by O'Dowd [5] in the British Medical Journal (BMJ) said that doctors, nurses, prison officers, and other staff would face a greater risk of assault if smoking were to be banned in such environments. Enforcing a complete blanket ban on smoking tobacco products in prisons could potentially create a bigger risk to staff and could result in potential riotous behaviour by prisoners, increase in injuries and assaults to staff, a view confirmed by the survey results presented here. The Irish Prison Smoking Policy states that there is such a value placed on cigarettes and tobacco products as a means of currency, that if they become contraband this would rival the existing drugs culture resulting in inmate discord causing an increase in the levels of assaults and violence amongst the prisoners themselves. Prisons have experienced riots when placing smoking bans into effect resulting in prisoners setting fires, destroying prison property, and persons being assaulted and injured. Such an incident occurred in Quebec (Canada) in February 2008, where a smoking ban enforced on 18 prisons was subsequently reversed following rioting by prisoners in these prisons.

The results of an inquiry on smoking bans in European prisons revealed that 22 (79%) out of 28 respondents (EU Member States plus Switzerland and Monaco) have introduced smoking bans in all of their prisons. The Irish Prison Officer Association (POA) health and safety coordinator Nigel Mallen claimed that the Government did not look hard enough to find ways of enforcing the ban in prisons. 3,150 members of the POA were prepared to challenge the constitutionality of the exemption of the ban in their workplaces. The working group appointed by the Director of Human Resources for the Irish Prison Service published a smoking policy in 2006 identifying prison recreational halls and circulation areas as the greatest risk of exposure to passive smoking for both staff and inmates. This working group also declared that limiting smoking to outside recreation yards and cellular accommodation may prove to be the most practical way to work towards a smoke-free prison environment. However, it was stated that this would still create major operational and management difficulties and therefore to minimise the impact, such restrictions would need to be implemented on a phased basis. Prison establishments or holding units for juveniles (persons under 18 years of age) must be entirely smoke-free environments, with smoking prohibited. Therefore, the policies adopted in such facilities should be applied to policies in all adult prisons.

Smoking bans in prisons must be implemented concurrently with cessation services appropriate for the client group which offer the prospect of long-term cessation. Better management of smoking in prisons should ensure that nonsmoker prisoners are not subjected to SHS in cells. In addition, nonsmoking prisoners need to be supported to prevent them starting smoking while in prison.

In England, two preventive programmes, the Acquitted programme and a nicotine replacement therapy programme were developed which offered prisoners group or one-to-one counselling and NRT with nicotine patches or Bupropion (Zyban), free of charge. An evaluation of the programmes between April 2004 and March 2005 in 16 prisons found the average quit rate for 4 weeks was 41%, validated by carbon monoxide monitoring. Results such as this highlight the huge potential in using smoking cessation programmes in prisons. However the results from this study show that prison officers are exposed to SHS in the workplace, and that 44% of nonsmoking prison officers have exhaled CO levels that would classify them as active smokers; these CO levels are slightly above those reported in Dublin bar staff before the smoking ban [6]. These results show that there is scope for improvement and to reduce staff exposure to SHS. This could potentially be achieved by having designated outdoor smoking areas.

### 9.1. SHS Particulate Exposures

In a study of ultrafine airborne particulates in 12 Dublin pubs prior to the workplace smoking ban coming into force, concentrations on occasions reached 250,000 per cm^3^ with an overall average of approximately 85,000 per cm^3^. Before the ban the typical concentrations in pubs were approximately 20,000 particles per cm^3^ with maximum values of around 80,000 per cm^3^ [7]. The results from this study in psychiatric hospitals above show the average ultrafine particle concentrations (130,000 per cm^3^) almost twice as high as the levels in a Dublin pub before the ban and 6.5 times higher than a Dublin pub in the postban period. This shows beyond any doubt that excessive SHS levels prevail in some of the psychiatric hospitals, and that both staff and residence are at risk of excessive exposures, and clearly warrant some significant changes in practices to protect staff and nonsmoking residents.

In comparing the PM_2.5_ results in the psychiatric hospitals to the results of a study of PM_2.5_ levels in Dublin pubs [6], psychiatric hospital levels (39.45 *μ*g/m^3^) are similar to the Dublin pubs before the introduction of the smoking ban (35.5 *μ*g/m^3^). However these PM_2.5_ levels in psychiatric hospitals (39.46 *μ*g/m^3^) are 8 times higher than the Dublin pub levels after the implementation of the ban (4.8 *μ*g/m^3^), showing that current levels in psychiatric hospitals are significantly greater that current levels in pubs. Again the results from the nursing homes (33 *μ*g/m^3^) are consistent with the levels observed in Dublin pubs prior to the smoking ban (35.5 *μ*g/m^3^).

For the purpose of putting these exposure levels in the nursing homes and psychiatric hospitals into context we compare them with levels measured by McLaughlin et al. [8, 9] and Hogg [10] within 33 Irish dwellings during 2005-2006. The results found that dwellings with smokers present had an average ultrafine particle level of 42,700 particles per cm^3^, while dwellings with only nonsmokers had an average ultrafine particle level of 16,500 particles per cm^3^. Comparing these results to the levels in the psychiatric hospitals and nursing homes, the average particle concentration in an exempted psychiatric hospital (130,000 per cm^3^) is more than 3 times the particle concentration found in selected Irish dwellings with smokers resident, and almost 8 times that of dwellings with only nonsmokers resident.

### 9.2. Nicotine Levels

Although we only present limited nicotine exposure data, there is clear evidence that nicotine levels in nursing home that allow smoking are significantly greater than in those where smoking is banned. Likewise the nicotine levels from the psychiatric hospitals and the nursing homes are extremely high and are consistent, if not higher than those from other published studies from pubs before smoking bans, [11–13].

## 10. Conclusion

This work has shown that in workplaces which are currently exempt from the workplace smoking ban, staff are clearly being exposed to ETS while doing their daily duties. The levels of exposure are quite varied depending on the individual workplace and practices; however in many cases they are seen to be similar to, or above those observed in Dublin pubs prior to the workplace smoking ban, and in addition current levels in these exempted areas are significantly above current levels in pubs. Clearly there is still significant room for improvement. As a party to the WHO FCTC, the Irish Government is under a legal obligation to take action on smoke-free environments. Staff working in exempted areas such as prisons, psychiatric hospitals, and nursing homes are unfairly exposed to SHS while in their place of work. While a complete smoking ban on indoor smoking in currently exempt premises may pose implementation challenges, staff should not be exposed to SHS while at work. Policies to ensure full protection of workers from SHS must be developed and implemented as a right for these workers and indeed inmates and patients.

## Figures and Tables

**Table 1:** Summary data table of exposure levels within 6 psychiatric hospitals.

	Ultrafine aerosol concentration (particles/cm^3^)			Particulate mass (*μ*g/m^3^)			Carbon monoxide (ppm)	Nicotine
	Min	Mean	Max	Mean (PM_2.5_)	Mean (PM_10_)	Mean (TSP)		
1(a)	35887	185977	378758	NA	NA	NA	NA	60.83 *μ*g/m^3^
1(b)	25021	215303	392541	NA	NA	NA	NA	133.28
2(a)	13474	80116	188233	31.11	38.83	47.34	2.8 ± 2.37	46.69
2(b)	4363	46094	112755	28.73	32.11	44.57	1.8 ± 0.69	131.91
3(a)	37530	89996	206483	30.9	39.54	46.2	3.4 ± 0.66	NA
3(b)	48340	92235	149599	28.35	33.95	40.00	4.5 ± 0.86	NA
3(c)	73504	211368	368491	51.67	64.16	85.14	NA	NA
4	35113	94614	184366	29.12	42.0	53.8	2.2 ± 0.73	NA
5(a)	132016	217488	298100	24.79	21.43	56.67	5.3 ± 0.97	NA
5(b)	41260	163680	335283	103.10	159.29	203.94	4.8 ± 1.33	NA
6	11845	34844	98763	27.33	11.29	40.83	3.1 ± 0.37	NA

Avg	41668.5	130156	246670	39.46	49.18	68.72	3.87	93.17

## References

[1] Afshari A, Matson U, Ekberg LE (2005). Characterization of indoor sources of fine and ultrafine particles: a study conducted in a full-scale chamber. *Indoor Air*.

[2] Morawska L, Jamriska M, Bofinger ND (1997). Size characteristics and ageing of the environmental tobacco smoke. *Science of the Total Environment*.

[3] (2007). *Impact of Smoking ban on Care Homes*.

[4] Butler T, Richmond R, Belcher J, Wilhelm K, Wodak A (2007). Should smoking be banned in prisons?. *Tobacco Control*.

[5] O’Dowd A (2005). US experience of smokefree prisons. *British Medical Journal*.

[6] Goodman P, Agnew M, McCaffrey M, Paul G, Clancy L (2007). Effects of the Irish smoking ban on respiratory health of bar workers and air quality in Dublin pubs. *American Journal of Respiratory and Critical Care Medicine*.

[8] McLaughlin J, Hogg C, Guo LY Ultrafine and coarse mode aerosol measurements in selected dwellings in Ireland.

[9] McLaughlin J, Hogg C The influence of ultrafine particles and occupancy factors on the risk from radon in some Irish dwellings.

[10] Hogg C (2006). *Ultrafine aerosol concentrations and their impact on radon progeny doses in selected Irish dwellings*.

[11] Mulcahy M, Evans DS, Hammond SK, Repace JL, Byrne M (2005). Secondhand smoke exposure and risk following the Irish smoking ban: an assessment of salivary cotinine concentrations in hotel workers and air nicotine levels in bars. *Tobacco Control*.

[12] Ellingsen DG, Fladseth G, Daae HL (2006). Airborne exposure and biological monitoring of bar and restaurant workers before and after the introduction of a smoking ban. *Journal of Environmental Monitoring*.

[13] Larsson M, Boëthius G, Axelsson S, Montgomery SM (2008). Exposure to environmental tobacco smoke and health effects among hospitality workers in Sweden—before and after the implementation of a smoke-free law. *Scandinavian Journal of Work, Environment and Health*.

